# Predicting conversational satisfaction of face-to-face conversation through interpersonal similarity in resting-state functional connectivity

**DOI:** 10.1038/s41598-024-56718-7

**Published:** 2024-03-12

**Authors:** Shigeyuki Ikeda, Hyeonjeong Jeong, Yukako Sasaki, Kohei Sakaki, Shohei Yamazaki, Takayuki Nozawa, Ryuta Kawashima

**Affiliations:** 1 Department of Ubiquitous Sensing, Institute of Development, Aging and Cancer, Tohoku University, Sendai, Japan; 2 RIKEN Center for Advanced Intelligence Project, Tokyo, Japan; 3 Graduate School of International Cultural Studies, Tohoku University, Sendai, Japan; 4 Department of Advanced Brain Science, Institute of Development, Aging and Cancer, Tohoku University, Sendai, Japan; 5 Department of Human Brain Science, Institute of Development, Aging and Cancer, Tohoku University, Sendai, Japan; 6 Research Institute for the Earth Inclusive Sensing, Tokyo Institute of Technology, Tokyo, Japan

**Keywords:** Face-to-face conversation, Interpersonal similarity, Resting-state functional connectivity, Cooperation, Neural decoding

## Abstract

When conversing with an unacquainted person, if it goes well, we can obtain much satisfaction (referred to as conversational satisfaction). Can we predict how satisfied dyads will be with face-to-face conversation? To this end, we employed interpersonal similarity in whole-brain resting-state functional connectivity (RSFC), measured using functional magnetic resonance imaging before dyadic conversation. We investigated whether conversational satisfaction could be predicted from interpersonal similarity in RSFC using multivariate pattern analysis. Consequently, prediction was successful, suggesting that interpersonal similarity in RSFC is an effective neural biomarker predicting how much face-to-face conversation goes well. Furthermore, regression coefficients from predictive models suggest that both interpersonal similarity and dissimilarity contribute to good interpersonal relationships in terms of brain activity. The present study provides the potential of an interpersonal similarity approach using RSFC for understanding the foundations of human relationships and new neuroscientific insight into whether success in human interactions is predetermined.

## Introduction

When conversing with someone during a first encounter, individuals sometimes get along well with the other person and sometimes do not. If face-to-face conversation between unacquainted people goes well, they can get a lot of satisfaction from the conversation (henceforth referred to as “conversational satisfaction”). High conversational satisfaction is a starting point for a good personal relationship. In order to know the extent to which people gain conversational satisfaction from face-to-face conversation, at present, there is no other way but to try it. Can we predict how satisfied dyads will be during face-to-face conversation?

Like attracts like. For example, people who have similar personality traits are attracted to each other^1^, which is referred to as the similarity effect. In contradiction to the similarity effect, dyadic interactions in complementary partnerships give us more satisfaction than those with similar partners^2^. In addition, a previous study using a machine learning approach showed that the quality of future interpersonal relationships could be predicted based on personality traits^3^. Although it remains unclear whether similarity or dissimilarity is important in conversational satisfaction, these previous findings suggest that conversational satisfaction can be predicted based on dyadic personalities.

Although a prediction based on dyadic personalities is thought to be a valid approach, a prediction using neuroimaging data may be a better approach. Previous studies using functional magnetic resonance imaging (fMRI) have shown that resting-state functional connectivity (RSFC), which is computed from brain activity during rest and characterizes the nature of interactions between different brain areas^4^, is associated with individual differences, such as intelligence^5^, emotional intelligence^6^, empathy^7^, personality^8^, and many behavioral measures^9^. Furthermore, RSFC serves as a fingerprint that can identify individuals from a large group^10^. If RSFC reflects behavioral individual differences, interpersonal similarity in RSFC may exhibit interpersonal similarity in behavior. Actually, interpersonal similarity in RSFC was positively correlated with interpersonal similarity in personality^11^. In addition, interpersonal closeness in the social network could be predicted from interpersonal similarity in RSFC^12^; importantly, an association between interpersonal closeness and interpersonal similarity in personality was not observed. This suggests that interpersonal similarity in RSFC contributes more to predicting conversational satisfaction than personality. A recent study investigated whether compatibility between heterosexual individuals could be predicted using RSFC^13,14^. More specifically, opposite-sex pairs talked to each other only once, and a pair was labeled as compatible if they wanted to talk to each other again; pairs were successfully classified into compatible and incompatible pairs based on RSFC contrast computed from pairs. These previous findings imply that interpersonal similarity in RSFC is a promising approach for predicting conversational satisfaction.

The present study had four aims. First, we investigated whether conversational satisfaction could be predicted using interpersonal similarity in RSFC. In particular, subjects underwent resting-state fMRI. After that, they completed face-to-face conversations with same-sex counterparts (i.e., in order to prevent romantic relationships from affecting conversational satisfaction) and rated the subjective satisfaction they gained from the conversation. Using multivariate pattern analysis, we created a predictive model and tested if the model could predict conversational satisfaction from interpersonal similarity in RSFC. Commonly, interlocutors gain and update each other’s information through initial conversation (e.g., identification of things that are in common and increased ability to predict the behavior of a partner), which can affect one’s conversational satisfaction gained from subsequent conversation. If conversational satisfaction changes through the repetition of conversation, a simple question arises, that is, does the prediction of conversational satisfaction go well in both initial and subsequent conversation? In summary, our first aim was to investigate whether not only satisfaction gained from initial conversation but also that from subsequent conversation could be predicted using interpersonal similarity in RSFC. Second, in order to confirm superiority of the interpersonal similarity approach using RSFC, we investigated whether interpersonal similarity in RSFC outperformed interpersonal similarity in personality (the Big Five personality traits) in terms of predicting conversational satisfaction. Third, dyads can have different perceptions of satisfying conversation, that is, dyadic conversational satisfaction does not always correspond with both individuals, for instance, a case in which one person is satisfied with a conversation but the other is dissatisfied with it. We investigated whether interpersonal similarity in RSFC has information related to interpersonal agreement of conversational satisfaction, using a prediction approach. Finally, it still remains unclear whether interpersonal similarity or dissimilarity contributes to good interpersonal relationships. We sought to investigate this in terms of brain activity. The present study emphasized the effectiveness of the interpersonal similarity approach in resting-state fMRI. The findings could contribute toward understanding the neural substrates underlying face-to-face conversation satisfaction.

## Results

### Procedure

**Figure 1:**
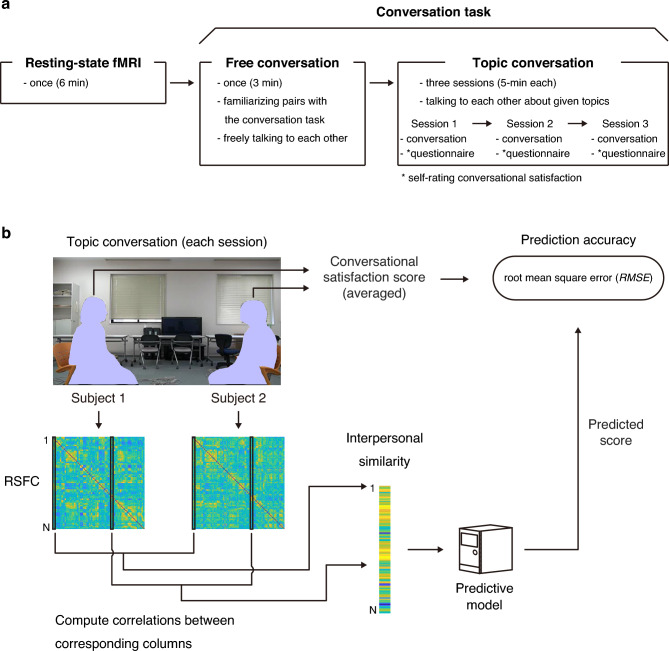
Graphical overview of the experiment and prediction analysis procedure using interpersonal similarity in resting-state functional connectivity (RSFC). (**a**) After undergoing resting-state fMRI, each pair of subjects completed the conversation task consisting of free conversation and topic conversation. After each of the topic conversation sessions, individual subjects in each pair completed a questionnaire to evaluate subjective satisfaction gained from the conversation. (**b**) RSFC was calculated from the resting-state fMRI data using Pearson correlation coefficients. For each pair, interpersonal similarity in RSFC was then determined using Pearson correlation coefficients between corresponding columns in two RSFC matrices (note that diagonal elements were excluded). In each step of a tenfold cross-validation procedure, a predictive model was then trained on training data (26 or 27 pairs) and tested on testing data (2 or 3 pairs). Thereafter, prediction accuracy was estimated by calculating the root mean square error (RMSE) between observed and predicted conversational satisfaction scores. In order to compute a *p* value of an RMSE value, we performed a permutation test (10,000 times). The threshold for statistical significance was set at *p* < 0.05.

An experimental overview is shown in Fig. 1a. After the completion of six-min resting-state fMRI scanning, all 29 pairs (15 male-male and 14 female-female pairs) were assigned to complete the conversation task consisting of free conversation and three topic-conversation sessions in a natural setting (see Conversation task section). The two subjects in each pair assessed conversational satisfaction using an eight-point Likert scale after each topic-conversation session. The two satisfaction scores for each individual pair were averaged, resulting in 29 conversational satisfaction scores for each topic-conversation session.

A schematic illustration of the prediction analysis procedure using interpersonal similarity in RSFC is shown in Fig. 1b. We performed standard fMRI preprocessing on the resting-state fMRI data (see Image Acquisition and fMRI Preprocessing sections). To calculate an RSFC matrix at the individual level (see RSFC section), the whole brain was parcellated into 120 regions using anatomical parcellation (AAL2; automated anatomical labeling). In addition to AAL2, we used the data-driven parcellation method (i.e., group ICA; group spatial independent component analysis, see RSFC section for details) to assess prediction accuracy in multiple parcellation methods. Resting-state fMRI time series for individual regions were calculated by averaging the voxel-wise time series within individual regions. We calculated the Pearson correlation coefficient between the averaged time series for each pair of regions, resulting in a 120 × 120 RSFC matrix. Interpersonal similarity in RSFC was then estimated by calculating the Pearson correlation coefficients between corresponding columns in a pair of RSFC matrices (see Interpersonal similarity in RSFC section). To estimate the prediction accuracy of conversational satisfaction from interpersonal similarity in RSFC, a tenfold cross-validation procedure was performed (see Prediction of conversational satisfaction section). In each cross-validation step, a predictive model was created using training data (26 or 27 pairs) with a support vector regression (SVR) algorithm, to predict the left-out conversational satisfaction from the testing data (2 or 3 pairs). Note that in addition to the SVR, we employed a ridge regression algorithm to evaluate prediction accuracy in the multiple regression algorithms. The root mean square error (RMSE) between observed and predicted conversational satisfaction was used to assess predictive power. The cross-validation procedure was then repeated 20 times to assess the sensitivity of prediction accuracy to different fold splits. The statistical significance of prediction accuracy was tested using a permutation test (*p* < 0.05).

### Observed conversational satisfaction

**Figure 2:**
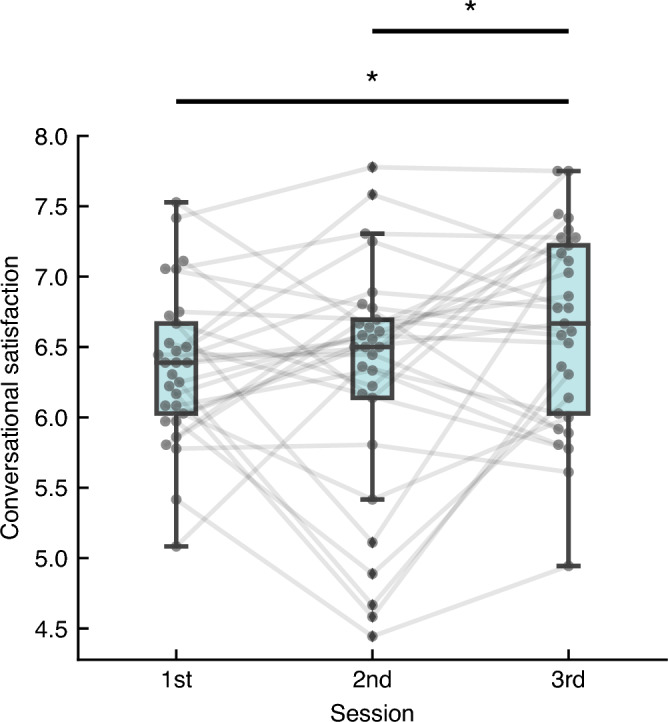
Conversational satisfaction observed in the three topic conversation sessions. In each boxplot, the center line and extent represent the median and interquartile range (IQR; 25th–75th percentile). The whiskers extend to 1.5 × IQR. Individual boxplots are overlaid with conversational satisfaction of 29 pairs. Each pair’s conversational satisfaction across the three sessions is connected with a gray line. Differences in means across the sessions were assessed using one-way repeated-measures analysis of variance and post-hoc analysis (*p* < 0.05 corrected for multiple comparisons using Shaffer’s method).

The conversational satisfaction observed in the three topic-conversation sessions is shown in Fig. 2 (see Supplementary Fig. 1 for conversational satisfaction for individual subjects). All the sessions exhibited relatively high medians of conversational satisfaction (i.e., > 6). The tendency was consistent with a previous report^15^. To investigate whether conversational satisfaction changed across the sessions, we performed a one-way repeated-measures analysis of variance (within-subject factor: session) using the R function “anovakun” (v.4.8.4) in R (v.3.3.2). The analysis showed a significant difference between the sessions (*F*_(2, 56)_ = 3.74, *p* = 0.03, $\eta_{{\text{p}}}^{2}$ = 0.12, $\eta_{{\text{G}}}^{2}$ = 0.043). Post-hoc analysis revealed that conversational satisfaction in the third session was higher relative to that observed in the first and second sessions (*p* < 0.05 corrected for multiple comparisons using Shaffer’s method). These results indicated that conversational satisfaction changed through repetition of conversation.

### Prediction results of conversational satisfaction

**Figure 3:**
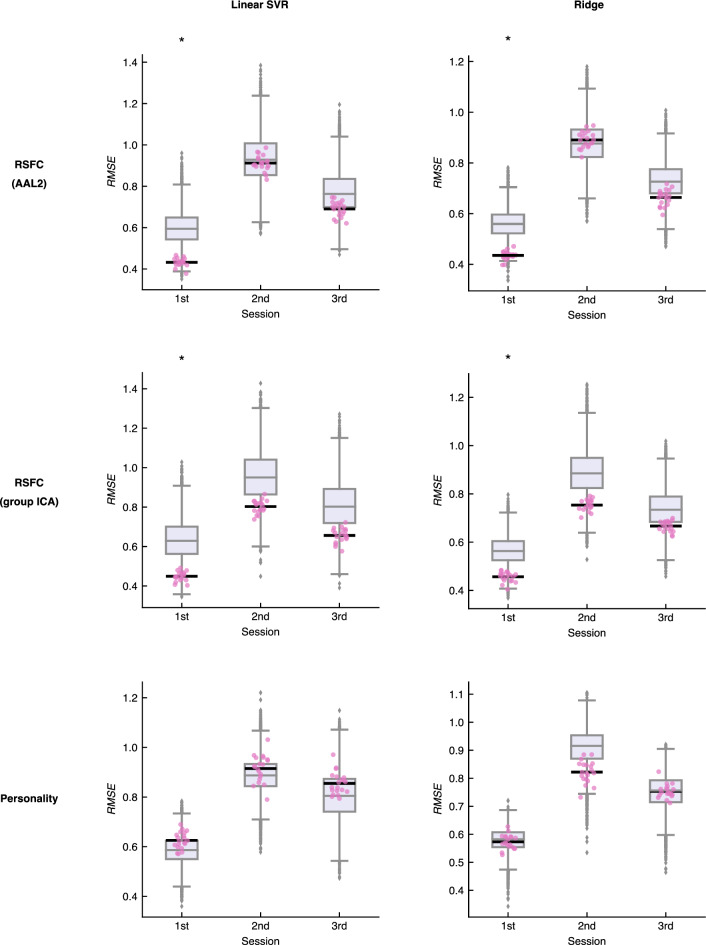
Prediction accuracy of conversational satisfaction. Predictive models were trained to predict conversational satisfaction from interpersonal similarity in RSFC or personality. Prediction accuracy was assessed using RMSE between observed and predicted conversational satisfaction. Individual dots (pink) show an RMSE value obtained from each of 20 iterations of the tenfold cross-validation. Each horizontal black line shows the mean accuracy of 20 iterations. Individual boxplots show the null distribution of prediction accuracy obtained by the permutation test (10,000 times). In each boxplot, the center line and extent represent the median and interquartile range (IQR; 25th–75th percentile). The whiskers extend to 1.5 × IQR. Each asterisk shows the statistical significance of mean prediction accuracy (i.e., horizontal black line). *Linear SVR* Linear support vector regression, *Ridge* Ridge regression, *RSFC* Resting-state functional connectivity, *AAL2* Automated anatomical labeling, *Group ICA* Group spatial independent component analysis, *RMSE* Root mean square error.

We examined whether conversational satisfaction of individual pairs could be predicted using interpersonal similarity in RSFC (Fig. 3). Interpersonal similarity in RSFC computed using AAL2 showed significant prediction accuracy in the first session (Linear SVR: *RMSE* = 0.43, *p* = 0.0083; Ridge: *RMSE* = 0.44, *p* = 0.011), while it did not show significant prediction accuracy in the second (Linear SVR: *RMSE* = 0.91, *p* = 0.44; Ridge: *RMSE* = 0.89, *p* = 0.57) or third sessions (Linear SVR: *RMSE* = 0.69, *p* = 0.22; Ridge: *RMSE* = 0.66, *p* = 0.18). Similarly, interpersonal similarity in RSFC computed using group ICA showed significant prediction accuracy in the first session (Linear SVR: *RMSE* = 0.45, *p* = 0.023; Ridge: *RMSE* = 0.46, *p* = 0.028), but it did not show significant prediction accuracy in the second (Linear SVR: *RMSE* = 0.80, *p* = 0.12; Ridge: *RMSE* = 0.75, *p* = 0.064) or third sessions (Linear SVR: *RMSE* = 0.66, *p* = 0.11; Ridge: *RMSE* = 0.67, *p* = 0.19). These results were consistent in the multiple regression methods (i.e., SVR and ridge regression) and multiple parcellation methods (i.e., AAL2 and group ICA), suggesting that interpersonal similarity in RSFC can predict conversational satisfaction obtained from the first conversation, but cannot predict changed conversational satisfaction in subsequent conversations. On the other hand, we investigated whether interpersonal similarity in personality could predict conversational satisfaction (see Interpersonal similarity in personality traits section). Interpersonal similarity in personality showed no significant prediction accuracy in all the sessions and regression algorithms, that is, the first session (Linear SVR: *RMSE* = 0.63, *p* = 0.76; Ridge: *RMSE* = 0.57, *p* = 0.41), second session (Linear SVR: *RMSE* = 0.91, *p* = 0.66; Ridge: *RMSE* = 0.82, *p* = 0.10), and third session (Linear SVR: *RMSE* = 0.86, *p* = 0.69; Ridge: *RMSE* = 0.75, *p* = 0.48). These results suggest that interpersonal similarity in RSFC yields better predictive power than interpersonal similarity in personality.

As a supplementary analysis, we investigated whether conversational satisfaction in the first session could be affected by subjects’ demographic characteristics and conversational topics used in the session. Specifically, we performed multiple linear regression analysis using ordinary least squares, in which conversational satisfaction in the first session was included as a dependent variable, and the following variables, that is, sex, mean age, age gap, department, and topic were included as independent variables (Supplementary Table 1). The analysis showed no significant independent variables when a threshold (uncorrected *p* < 0.05, two-tailed) was applied (Supplementary Table 2). This indicated that conversational satisfaction in the first session was weakly affected by the subjects’ demographic characteristics and conversational topics.

### Prediction results of interpersonal agreement of conversational satisfaction

We investigated whether interpersonal similarity in RSFC or personality could predict interpersonal agreement of conversational satisfaction in each pair (Supplementary Fig. 2). Specifically, interpersonal agreement of conversational satisfaction was defined as an absolute difference between two conversational satisfaction scores obtained from each pair of subjects. If the absolute difference is a zero, it means that subjects of its pair are in complete agreement about conversational satisfaction. The absolute difference served as a target for prediction using interpersonal similarity. The prediction procedure in this analysis was identical with that used in prediction of conversational satisfaction (see Prediction of conversational satisfaction section). As a result, we found no significant prediction accuracy.

### Regression coefficients and permutation feature importance

Here, we examined whether interpersonal similarity or dissimilarity was important in a face-to-face conversation context. To this end, we extracted regression coefficients from predictive models showing significant prediction accuracy (see Regression coefficients of a predictive model section). Individual regression coefficients represent a relationship between individual columns of an interpersonal similarity matrix and conversational satisfaction. If a regression coefficient is a positive value, it represents a positive correlation between interpersonal similarity and conversational satisfaction. As a result, we observed regression coefficients deviating from medians of regression coefficients from the null predictive models. Regarding AAL2 (Supplementary Fig. 3), a visual inspection of the regression coefficients suggested seven regions deviating from medians of null regression coefficients, that is, the superior frontal gyrus (i.e., Frontal_Sup_2_L), rectus (i.e., Rectus_R), inferior occipital gyrus, (i.e., Occipital_Inf_L), supramarginal gyrus (i.e., SupraMarginal_R), caudate (i.e., Caudate_L), and cerebellum (i.e., cerebelum_3_R and Vermis_9). Of these regression coefficients, three coefficients had positive values, while four had negative values. On the other hand, regarding group ICA (Supplementary Fig. 4), the visual inspection of regression coefficients suggested nine networks deviating from medians of the null regression coefficients, that is, the sensorimotor networks (IC 1, 8, 23, and 30), primary visual network (IC 14), ventral default mode network (IC 15), precuneus network (IC 34), executive control network (IC 42), and dorsal default mode network (IC 58). Of these regression coefficients, four coefficients had positive values, while five had negative values. These results suggest that both interpersonal similarity and dissimilarity increase conversational satisfaction.

In addition to regression coefficients, we computed permutation feature importance (henceforth referred to as permutation importance) for each feature (see Permutation importance section) to investigate the extent to which individual features (i.e., each column of an interpersonal similarity matrix) contributed to the prediction of conversational satisfaction. We used RMSE as a metric for prediction accuracy, so that smaller permutation importance, that is, a negative value, showed that their features contributed more to the prediction. As a result, we observed permutation importance deviating from the medians of permutation importance from the null predictive models. Regarding AAL2 (Supplementary Fig. 5), a visual inspection of permutation importance suggested six regions deviating from the medians of null permutation importance, that is, the superior frontal gyrus (i.e., Frontal_Sup_2_L), rectus (i.e., Rectus_R), inferior occipital gyrus, (i.e., Occipital_Inf_L), supramarginal gyrus (i.e., SupraMarginal_R), caudate (i.e., Caudate_L), and cerebellum (i.e., cerebelum_3_R). Next, regarding group ICA (Supplementary Fig. 6), the visual inspection of permutation importance suggested eight networks deviating from medians of null permutation importance, that is, the sensorimotor networks (IC 1, 8, 23, and 30), primary visual network (IC 14), ventral default mode network (IC 15), precuneus network (IC 34), and executive control network (IC 56). These regions and networks virtually agreed with those observed in regression coefficients, which corroborated the implication from visual inspection of the regression coefficients.

### Prediction results of conversational satisfaction from respective subjects of individual pairs

So far conversational satisfaction from individual pairs in the first session could be predicted using interpersonal similarity in RSFC. This leads to a simple question, that is, could individual subjects’ conversational satisfaction in each pair be predicted from interpersonal similarity in RSFC? Hence, we performed an additional prediction analysis (see Supplementary Fig. 7 for details). As a result, interpersonal similarity in RSFC computed using AAL2 showed significant prediction accuracy only in S2. The results suggest the difficulty in predicting individual subjects' conversational satisfaction from interpersonal similarity in RSFC.

## Discussion

Examination of resting-state brain activities in two brains could lead to new neuroscientific insight into the extent to which success and failure in human interactions are predetermined. The present study had four purposes. First, we aimed to predict how satisfied dyads, consisting of unacquainted people, will be with face-to-face conversation using their resting-state brain activities measured before the conversation. The prediction worked well for satisfaction gained from initial conversation but not from subsequent conversation. The results suggest that interpersonal similarity can be used as an effective neural biomarker to predict how satisfied dyads will be with initial conversation, but cannot predict conversational satisfaction from subsequent conversation. Second, we investigated whether interpersonal similarity in RSFC outperformed interpersonal similarity in personality with regard to predicting conversational satisfaction. We found the significant prediction accuracy only in RSFC, and the results were supported by previous findings^12^. Third, we investigated whether interpersonal agreement of conversational satisfaction could be predicted from interpersonal similarity in RSFC or personality. However, no significant prediction accuracy was observed. Finally, we investigated whether interpersonal similarity or dissimilarity contributes to good interpersonal relationships. The visual inspection of regression coefficients from predictive models suggests that either interpersonal similarity or dissimilarity in RSFC does not contribute to good interpersonal relationships, but rather that both similarity and dissimilarity are contributive. Our study highlights the value of resting-state brain activity for understanding the foundations of human relationships.

Interpersonal similarity in RSFC (AAL2 and group ICA) showed significant prediction accuracy in the first session but not in the subsequent sessions. These prediction results may stem from differences in conversation between the first and subsequent sessions. That is, initial face-to-face conversation primarily depends on each other’s behavioral phenotypes because pairs do not have each other’s information, and subsequent conversation depends on not only their behavioral phenotypes but also the information that one another gained from previous conversation. The observed conversational satisfaction could be indirect evidence for supporting the above inference. In particular, conversational satisfaction in the third session was higher than that of the first and second sessions, suggesting that gaining and updating each other’s information through conversation (e.g., identification of things in common and increased ability to predict behavior of a partner) resulted in greater conversational satisfaction. On the other hand, although an increase in conversational satisfaction was not observed between the first and second sessions, conversational satisfaction of individual pairs appeared to vary between the two sessions. This suggests that gaining and updating each other’s information did not necessarily increase conversational satisfaction, but caused changes in conversational satisfaction. Taken together, each other’s information gained from earlier conversation could influence conversational satisfaction of subsequent conversation, and the influence of such information might not have been reflected in resting-state brain activity measured before the conversation task. Therefore, conversational satisfaction in the first session could be predicted from interpersonal similarity in RSFC, which is believed to reflect interpersonal similarity in how people think and behave (i.e., behavioral phenotypes). On the other hand, conversational satisfaction in the subsequent sessions could not be predicted because interpersonal similarity in RSFC could not track the influence of each other’s information gained from previous sessions.

Conversational satisfaction could not be predicted using interpersonal similarity in personality, suggesting that interpersonal similarity in RSFC outperformed interpersonal similarity in personality when predicting conversational satisfaction. The results were supported by a previous study^12^; the study succeeded in predicting interpersonal closeness in the social network using interpersonal similarity in RSFC, and did not observe an association between interpersonal closeness and interpersonal similarity in personality. Our results suggest the superiority of the interpersonal similarity approach using RSFC. On the other hand, there was a previous study reporting that the quality of future interpersonal relationships could be predicted based on personality traits^3^. The previous findings were inconsistent with our prediction results obtained using interpersonal similarity in personality. The previous study focused on a long-term heterosexual relationship (i.e., four years later), while the present study focused on beginning of a relationship formed through face-to-face conversation with same-sex counterparts. These differences may have caused the inconsistency with previous findings. At least in our study, interpersonal similarity in personality did not go well.

Interpersonal agreement of conversational satisfaction could not be predicted using interpersonal similarity in RSFC. An absolute difference between two conversational satisfaction scores of each pair was used as an indicator of interpersonal agreement. When using the absolute difference, we assume that subjects with closer conversational satisfaction scores should be more similar to each other. For example, a pair of subjects whose scores are 1 and 2 is quite identical to a pair of subjects whose scores are 7 and 8 because their absolute difference is 1. However, in these cases, the mean of conversational satisfaction scores of one pair (i.e., (1 + 2)/2 = 1.5) is substantially different from that of another pair (i.e., (7 + 8)/2 = 7.5). In our prediction analysis, the mean conversational satisfaction could be predicted by interpersonal similarity in RSFC; that is, interpersonal similarity in RSFC is associated with mean conversational satisfaction. Therefore, the use of interpersonal similarity in RSFC may make it difficult to predict interpersonal agreement of conversational satisfaction because there are cases in which absolute differences are same even if mean values are different.

We examined regression coefficients from predictive models that showed significant prediction accuracy (i.e., the first session). Consequently, several regression coefficients were found to be contributive in the prediction models. Of the regression coefficients, both positive and negative coefficients were almost the same in number. A positive regression coefficient represents a positive correlation between interpersonal similarity in RSFC and conversational satisfaction, and vice versa. Therefore, our study suggests that both interpersonal similarity and dissimilarity in RSFC contribute toward good interpersonal relationships. This was supported by a previous study, which succeeded in classifying opposite-sex pairs into compatible and incompatible pairs using their RSFCs, and reported that both similarity and dissimilarity in RSFC between individuals contributed to compatibility classification^13,14^. On the other hand, the comparison of our study with the previous study leads to a simple question, that is, how does the difference in conversation between same-sex and opposite-sex pairs affect the prediction of conversational satisfaction using interpersonal similarity in RSFC? Further research will be required to tackle this question.

The visual inspection of regression coefficients (AAL2) suggested importance of the superior frontal gyrus, rectus (i.e., the orbitofrontal region), inferior occipital gyrus, supramarginal gyrus (including temporal-parietal junction), caudate (a part of the striatum), and cerebellar regions. All the regions excluding the cerebellar regions have been reported as important areas that show significant speaker–listener neural coupling^16^ (Note that the experimental setting in the previous study was different from conversation). Our results suggest that these regions play important roles in not only speaker-listener interactions but also successful face-to-face conversation. A previous study succeeded in classifying opposite-sex pairs into compatible and incompatible pairs using their RSFCs, and reported certain brain areas that significantly contributed to the compatibility classification^13,14^. These brain areas partially overlapped with those of our study (i.e., rectus, caudate, and cerebellar regions), which suggests the validity of our results.

The visual inspection of regression coefficients (group ICA) suggested the sensorimotor and default mode networks. The sensorimotor network is associated with not only sensorimotor function but also empathy. For example, the sensorimotor network is crucial for inferring amusement from another’s smile^17^. On the other hand, the default mode network overlaps with areas involved in social cognition^18^. In addition, the mentalizing network, which is associated with the cognitive process of inferring thoughts, feelings, or beliefs of other people, is characterized by regions of the default mode network^19^. Therefore, interpersonal similarity for the sensorimotor network or default mode network may reflect relationships between each other’s ability to infer the other person’s thoughts and feelings, suggesting that its relationships are important for satisfying conversation.

Because group ICA was performed on the entire dataset, our analysis procedure could have been biased. To avoid bias, data should ideally be divided into training and testing sets, and group ICA should be applied only to the training set. However, the procedure for group ICA did not use test labels (i.e., conversational satisfaction scores) at all. Therefore, there was no potential bias toward the prediction results. In addition, the analysis procedure was supported by a previous study^20^.

This study focused on resting-state brain activity for understanding the foundations of human relationships. On the other hand, hyperscanning, i.e., the simultaneous recording of the activity of multiple brains during social interactions, is a valid alternative to examine human relationships. A previous study on hyperscanning suggests that the neural synchronization between partners may underlie successful face-to-face communication^21^. Therefore, combination of interpersonal similarity in RSFC and neural synchronization may enhance prediction accuracy of conversational satisfaction.

The study was subject to several limitations. First, we inferred that conversation after the first session depends on both each other’s behavioral phenotypes and information gained from previous conversation. However, the only evidence for supporting its inference was the observed conversational satisfaction, and it was not direct evidence. In future research, additional subjective questionnaires will be needed to assess the influence of partners’ information on conversations. Second, the sample size in this study was small relative to that needed for individual differences research^22^. Establishing the reliability and reproducibility of our results is an important challenge for future research. To overcome the small sample size problem, a previous approach may be helpful^13,14^. In the approach, individual subjects converse with every other subject. Third, the RSFC-based predictive model had 120 or 62 features and the personality-based model had 5 features. As such, the difference in the number of features may make the comparison between the two models unfair. The visual inspection of permutation importance suggested six AAL2 regions (and eight networks in group ICA) contributing to the prediction of conversational satisfaction. A reduced prediction model created from the regions (and the networks) may allow for fair comparison with the personality-based model. However, to perform the comparison, another dataset is needed. Fourth, although we employed Pearson correlation coefficient as a measure to compute interpersonal similarity in RSFC, there are some alternative measures that can be employed, namely cosine similarity. Thus, comparing Pearson correlation coefficients with other measures may be intriguing in prediction accuracy. Lastly, the subjects in the present study were all young, healthy university or postgraduate students. Further research is required to confirm whether the current findings hold true across different age groups and educational levels.

In conclusion, the present study demonstrated that interpersonal similarity in RSFC served as a neural biomarker for predicting the extent to which interlocutors gain conversational satisfaction from face-to-face conversation. Furthermore, our prediction analysis indicated several regression coefficients contributing to the prediction of conversational satisfaction; both positive and negative coefficients existed in these regression coefficients. These results suggest that in terms of brain activity, both interpersonal similarity and dissimilarity contribute to good interpersonal relationships. The interpersonal similarity approach using RSFC provides not only the value of resting-state brain activity for understanding the foundations of human relationships but also new neuroscientific insight into the extent to which the success of human interactions is predetermined.

## Methods

### Subjects

The present study was approved by the Ethics Committee at Tohoku University Graduate School of Medicine, and was performed in accordance with the Declaration of Helsinki. The subjects included 58 Japanese, healthy, right-handed university or postgraduate students (30 men and 28 women, mean age: 20.8 ± 1.4 years) with no history of psychiatric illness. We obtained written informed consent from all subjects. Subjects were recruited on the Tohoku University campus using email and bulletin boards, and were screened for criteria to ensure that they were healthy, right-handed, and had no history of psychiatric illness. All subjects completed a self-report questionnaire in which they indicated their sex, age, and departments at the university. In addition, the subjects completed a Japanese questionnaire, the “Big Five Scales,” developed in a previous study^23^, to assess their personality traits. The questionnaire was constructed based on the Adjective Check List^24^ and consists of 60 items (12 items per trait). After completing the questionnaires, the subjects were randomly assigned to pairs (29 pairs; 15 male-male and 14 female-female pairs). The subjects did not know each other. All pairs underwent resting-state fMRI scanning followed by a conversation task.

Aside from the above-mentioned subjects, 35 subjects (17 men and 18 women, mean age: 21.7 ± 1.7 years) who were recruited without criteria for participation completed a questionnaire to assess their ease in talking about each of 18 preliminarily chosen topics. Each topic was rated using a seven-point Likert scale. The top three topics (i.e., my hobbies, school life, and travel) were selected from the 18 topics based on rating scores (Supplementary Table 3). All 35 subjects had undergone another psychological study that was unrelated to our study before completing the questionnaire. The psychological study will not be described here.

### A conversation task

After the completion of resting-state fMRI scanning, the subject pairs completed a free-conversation session followed by three topic-conversation sessions (Fig. 1a). In the free-conversation session, individual pairs were instructed to talk freely to each other for three minutes. The purpose of the session was to familiarize the pairs with the conversation task in a natural setting. In each topic-conversation session, individual pairs were instructed to talk to each other about a given topic for five minutes. One of the three topics was used randomly in each topic-conversation session; every topic was used once. After each topic-conversation session, the subjects in each pair completed a Japanese questionnaire measuring their subjective satisfaction with the conversation (i.e., conversational satisfaction). The questionnaire was derived from a rapport criterion measurement developed in a previous study^25^ and consists of 18 items (e.g., I was able to regulate conversation well and I was bored with conversation) rated on an eight-point Likert scale ranging from 1 (Strongly disagree) to 8 (Strongly agree)^15^. A conversational satisfaction score for each subject was calculated by averaging the 18 item scores. To calculate a conversational satisfaction score for each pair in each session, two conversational satisfaction scores for two subjects in each pair were averaged in every session.

### Image acquisition

Imaging data were collected using a 3-T Philips Intera Achieva scanner equipped with an 8-channel head coil. Resting-state fMRI images were acquired using an echo planar imaging sequence (64 × 64 matrix, TR = 2,000 ms, TE = 30 ms, flip angle = 80°, FOV = 192 mm, slice thickness = 3.5 mm, slice gap = 0.5 mm, and 32 transverse slices per volume). In the resting-state fMRI, the subjects were instructed to remain still, relax with their eyes closed, and remain awake and not to think about anything in particular. A total of 180 functional volumes were acquired for individual subjects. Whole-brain T1-weighted structural images for individual subjects were acquired according to the following parameters: 240 × 240 matrix, TR = 6.6 ms, TE = 3.0 ms, flip angle = 8°, FOV = 240 mm, slice thickness = 1.0 mm, and 162 transverse slices. During scanning, scanner noise was reduced using ear plugs, and subjects’ head motion was minimized via pads and Velcro tape.

### fMRI preprocessing

The fMRI image preprocessing was performed using a Statistical Parametric Mapping software (SPM12; Wellcome Department of Cognitive Neurology, London, UK, https://www.fil.ion.ucl.ac.uk/spm/) running under MATLAB R2016a (The MathWorks Inc., Natick, MA). The raw functional volumes were slice-timing-corrected for individual subjects, spatially realigned to the mean fMRI volume to correct for head motion artifacts, and unwarped to correct for movement-induced distortions. The structural T1 images for individual subjects were co-registered to the mean fMRI volumes for corresponding subjects and segmented to extract white matter (WM) and cerebrospinal fluid (CSF). These tissue maps were resliced to the mean fMRI volume and thresholded using a voxel-level threshold of 0.99 to create subject-specific masks for WM and CSF. We used the masks to extract mean time series from WM and CSF. To remove the effects of head motion and nonneuronal BOLD fluctuations, 27 nuisance covariates, including a linear trend, Friston 24 motion parameters^26^, and mean time series from WM and CSF, were regressed out from individual voxel time series. Furthermore, low-frequency noise was removed using a temporal high-pass filter with a cutoff frequency of 0.01 Hz. The subsequent fMRI volumes were normalized to the standard space of the Montreal Neurological Institute template using the forward deformation field derived from the segmentation process for the structural T1 image. The normalized fMRI volumes were smoothed using a 6-mm full-width at half-maximum Gaussian kernel.

### Resting-state functional connectivity (RSFC)

To estimate RSFC at an individual level, all fMRI volumes were divided into 120 regions based on an anatomical parcellation (i.e., AAL2)^27^. The representative time series in individual regions were calculated by averaging the fMRI time series over all voxels in individual regions. We calculated Pearson correlation coefficient between the averaged time series of each pair of regions, resulting in a 120 × 120 symmetrical RSFC matrix.

In addition to the anatomical parcellation, we employed a data-driven parcellation. In the parcellation, we used group ICA of the fMRI toolbox software v4.0b (GIFT, http://trendscenter.org/software/)^28^ running under MATLAB R2016a. Group ICA was performed for the preprocessed fMRI volumes for all 58 subjects after applying the AAL2 anatomical mask to the fMRI data. Details regarding the group ICA procedure are described below. Initially, we performed two data reduction steps^28^. In the first step, the preprocessed 180 fMRI volumes for individual subjects were reduced to 90 principal components via principal components analysis (PCA); in the second step, the reduced data from all subjects were concatenated and reduced to 70 principal components using PCA. The Infomax ICA algorithm^29^ was then performed 100 times in Icasso^30^, and aggregate spatial maps were acquired. Subject-specific time series for individual spatial maps were reconstructed using the GICA algorithm^28^. Consequently, 70 spatial maps were extracted using group ICA. The number of spatial maps was chosen, because it has previously been shown to produce resting-state networks comprising refined cortical and subcortical components^31,32^. However, we characterized a subset of 62 spatial maps as meaningful resting-state networks by removing physiological, motion, and imaging artifacts based on previous findings^33^. For individual subjects, a 62 × 62 symmetrical RSFC matrix was created by calculating the Pearson correlation coefficient between the time series of each pair of resting-state networks.

### Interpersonal similarity in resting-state functional connectivity (RSFC)

To estimate interpersonal similarity in RSFC, we calculated the Pearson correlation coefficients between RSFC values for each pair based on a previous study^34^. In particular, the Pearson correlation coefficients were calculated between correlation vectors of corresponding columns in two RSFC matrices for individual pairs (Fig. 1), creating an interpersonal similarity matrix (29 pairs × *N*); *N* represents the number of regions or networks (i.e., 120 [AAL2] or 62 [group ICA]). The interpersonal similarity matrix was used as input data for the prediction algorithm. Each row of the interpersonal similarity matrix was a vector of which individual elements represented interpersonal similarity computed for every region or network.

### Interpersonal similarity in personality traits

To assess subjects’ personality traits, we used a Japanese questionnaire, the “Big Five Scales” (see Subjects section). The questionnaire consists of 60 items (12 items per each of five traits). All subjects completed the questionnaire before resting-state fMRI scanning. Interpersonal similarity for individual pairs was assessed by calculating the Euclidean distances between subjects’ 12 scores for individual traits, resulting in an interpersonal similarity matrix (29 pairs × 5 traits). We used Euclidean distance, as it can be used to measure differences in mean scores, while the Pearson correlation coefficient is invariant to such differences.

### Prediction of conversational satisfaction

A linear epsilon SVR was used to predict conversational satisfaction scores from interpersonal similarity. The SVR algorithm was implemented using the scikit-learn Python package with default parameters^35^; the implementation of the SVR algorithm is based on the LIBSVM toolbox^36^. In addition to the SVR, we used ridge regression with default parameters to assess prediction accuracy in multiple common regression algorithms. The interpersonal similarity matrix was entered into each of the SVR and ridge regression to predict conversational satisfaction. A tenfold cross-validation procedure was performed to produce accuracy estimates. In each fold, a predictive model created from data consisting of 26 pairs (or 27 pairs) was used to predict conversational satisfaction for each of 3 pairs (or 2 pairs) based on its interpersonal similarity vector. Prediction accuracy was evaluated by RMSE between the observed and predicted scores. The cross-validation procedure was then repeated 20 times to assess the sensitivity of prediction accuracy to different fold splits. It is unknown which null distribution is associated with prediction accuracy. Therefore, a permutation test was performed to assess the statistical significance of prediction accuracy. Specifically, the permutation test was performed by randomly permuting all conversational satisfaction scores but preserving the structure of the interpersonal similarity matrix, and the repeated tenfold cross-validation was then performed on the new data. The entire procedure was repeated 10,000 times. Then, a* p* value corresponding to the actual RMSE (i.e., the mean of the 20 prediction accuracy values from the repeated cross-validation) was defined as the probability of observing RMSE values equal to or less than the actual RMSE. The threshold for statistical significance was set at *p* < 0.05.

### Regression coefficients of a predictive model

To investigate whether interpersonal similarity or dissimilarity contributes to good interpersonal relationships, we extracted regression coefficients from predictive models that were trained using conversational satisfaction in the first session and interpersonal similarity in RSFC (AAL2 or group ICA). In each step of the tenfold cross-validation procedure, a predictive model was created with the SVR algorithm, resulting in 10 predictive models. We then computed a mean predictive model by averaging the 10 predictive models. As the entire procedure was repeated 20 times, we obtained 20 mean predictive models. Finally, a mean predictive model was created by averaging the 20 mean predictive models. We focused on the regression coefficients of the mean predictive model.

To confirm importance in individual regression coefficients, we generated null models. In particular, we randomly permuted all conversational satisfaction scores, but preserved the structure of the interpersonal similarity matrix, and the repeated tenfold cross-validation was then performed on the new data. The entire procedure was repeated 10,000 times, resulting in 10,000 null models. We compared the regression coefficients of the mean predictive models with those of the null models.

### Permutation importance

To investigate the contribution of each input feature to the predictive model, permutation importance was computed using the scikit-learn Python package. In each step of the tenfold cross-validation procedure, a predictive model was created from training data consisting of 26 pairs (or 27 pairs) using the SVR algorithm, and the permutation importance was estimated using testing data consisting of 3 pairs (or 2 pairs). In particular, we computed the actual prediction accuracy *s* using the testing data, whose size was 3 × *N* or 2 × *N* (*N* = 120 [AAL2] or 62 [group ICA]); subsequently, each feature *j* (column of the testing data) was randomly shuffled to generate a corrupted version of the data. Prediction accuracy *s*_*k*,*j*_ was computed from the corrupted data. This procedure was repeated *K* times (= 5). Therefore, permutation importance *i*_*j*_ is defined as:

1 $$\begin{array}{*{20}c} {i_{j} = s - \frac{1}{K}\mathop \sum \limits_{k = 1}^{K} s_{k,j} } \\ \end{array}$$

We obtained ten permutation importance values of each feature through the tenfold cross-validation, and the mean permutation importance for each feature was computed by averaging the 10 importance values. The entire procedure was repeated 20 times (i.e., the repeated tenfold cross-validation), resulting in 20 mean permutation importance values for each feature. Finally, mean permutation importance was computed by averaging the 20 importance values. As RMSE was used as a metric for prediction accuracy, smaller permutation importance values (i.e., negative values) showed that their features were more important in prediction.

To investigate the significance of permutation importance for each feature, we computed the baseline of permutation importance by creating null models. In particular, we randomly permuted all conversational satisfaction scores, but preserved the structure of the interpersonal similarity matrix, and the repeated tenfold cross-validation was then performed on the new data. The entire procedure was repeated 100 times, resulting in 100 baseline importance values for each feature. We compared the actual permutation importance with the baseline importance.

### Supplementary Information

Supplementary Information.

## Data Availability

The datasets generated during and/or analyzed during the current study are not publicly available due to Institutional Review Board restrictions but are available from the corresponding author on reasonable request. Note that any requests must first be approved by the Ethics Committee at Tohoku University Graduate School of Medicine.

## Code availability

We performed most analyses using the custom code in MATLAB R2016a and Python (v.3.7.11). The custom code files are available upon request to the corresponding author.
