# Supplementary material for: Predicting conversational satisfaction of face-to-face conversation through interpersonal similarity in resting-state functional connectivity
Source: Sci Rep. 2024 Mar 12;14:6015. doi: 10.1038/s41598-024-56718-7 (PMC10933256; doi:10.1038/s41598-024-56718-7)
Supplement: Supplementary file 1 — Supplementary Information. [file 41598_2024_56718_MOESM1_ESM.docx]

**SUPPLEMENTARY MATERIALS**

**Supplementary Figure 1**

Conversational satisfaction for all subjects in individual sessions. Colored bars indicate conversational satisfaction from respective subjects of individual pairs. P = pair, S1 and S2 = a subject and the other subject in each pair

**Supplementary Figure 2**

Prediction accuracy of interpersonal agreement of conversational satisfaction. Predictive models were trained to predict interpersonal agreement from interpersonal similarity in RSFC or personality. Prediction accuracy was assessed using RMSE between observed and predicted interpersonal agreement. Individual dots (pink) show an RMSE value obtained from each of 20 iterations of the 10-fold cross-validation. Each horizontal black line shows the mean accuracy of 20 iterations. Individual boxplots show the null distribution of prediction accuracy obtained by the permutation test (10,000 times). In each boxplot, the center line and extent represent the median and interquartile range (IQR; 25th–75th percentile). The whiskers extend to 1.5 × IQR. Linear SVR = linear support vector regression, Ridge = ridge regression, RSFC = resting-state functional connectivity, AAL2 = automated anatomical labeling, group ICA = group spatial independent component analysis, RMSE = root mean square error

**Supplementary Figure 3**

Regression coefficients of a predictive model obtained using the SVR algorithm. The predictive model was trained using conversational satisfaction in the first session and interpersonal similarity in RSFC (AAL2), and showed significant prediction accuracy (i.e., top left of Figure 3). Individual pink dots show a mean regression coefficient of 20 iterations of 10-fold cross-validation (see Regression coefficients of a predictive model section). Individual boxplots show regression coefficients from null models that were created using randomly shuffled data (10,000 times). In each boxplot, the center line and extent represent the median and interquartile range (IQR; 25th–75th percentile). The whiskers extend to 1.5 × IQR. Region names are based on AAL2. SVR = support vector regression, RSFC = resting-state functional connectivity, AAL2 = automated anatomical labeling

**Supplementary Figure 4**

Regression coefficients of a predictive model obtained using the SVR algorithm. The predictive model was trained using conversational satisfaction in the first session and interpersonal similarity in RSFC (group ICA), and showed significant prediction accuracy (i.e., middle left of Figure 3). Individual pink dots show a mean regression coefficient of 20 iterations of the 10-fold cross-validation (see Regression coefficients of a predictive model section). Individual boxplots show regression coefficients from null models that were created using randomly shuffled data (10,000 times). In each boxplot, the center line and extent represent the median and interquartile range (IQR; 25th–75th percentile). The whiskers extend to 1.5 × IQR. Network names are based on the group ICA toolbox (GIFT). SVR = support vector regression, RSFC = resting-state functional connectivity, group ICA = group spatial independent component analysis, IC = independent component

**Supplementary Figure 5**

Permutation feature importance of a predictive model obtained using the SVR algorithm. The predictive model was trained using conversational satisfaction in the first session and interpersonal similarity in RSFC (AAL2), and showed significant prediction accuracy (i.e., top left of Figure 3). Individual pink dots show a mean permutation feature importance of 20 iterations of the 10-fold cross-validation (see permutation importance section). Individual boxplots show the permutation feature importance from null models that were created using randomly shuffled data (100 times). In each boxplot, the center line and extent represent the median and interquartile range (IQR; 25th–75th percentile). The whiskers extend to 1.5 × IQR. Region names are based on AAL2. SVR = support vector regression, RSFC = resting-state functional connectivity, AAL2 = automated anatomical labeling

**Supplementary Figure 6**

Permutation feature importance of a predictive model obtained using the SVR algorithm. The predictive model was trained using conversational satisfaction in the first session and interpersonal similarity in RSFC (group ICA), and showed significant prediction accuracy (i.e., middle left of Figure 3). Individual pink dots show a mean permutation feature importance of 20 iterations of the 10-fold cross-validation (see permutation importance section). Individual boxplots show the permutation feature importance from null models that were created using randomly shuffled data (100 times). In each boxplot, the center line and extent represent the median and interquartile range (IQR; 25th–75th percentile). The whiskers extend to 1.5 × IQR. Network names are based on the group ICA toolbox (GIFT). SVR = support vector regression, RSFC = resting-state functional connectivity, group ICA = group spatial independent component analysis, IC = independent component

**Supplementary Figure 7**

Prediction accuracy of individual subjects’ conversational satisfaction in the first session. As shown by Supplementary Figure 1, each pair was comprised of two subjects (i.e., S1 and S2). Using a linear support vector regression algorithm, predictive models were trained to predict conversational satisfaction of S1 or S2 from interpersonal similarity in RSFC. Prediction accuracy was assessed using RMSE between observed and predicted conversational satisfaction. Individual dots (pink) show an RMSE value obtained from each of 20 iterations of the 10-fold cross-validation. Each horizontal black line shows the mean accuracy of 20 iterations. Individual boxplots show the null distribution of prediction accuracy obtained by the permutation test (10,000 times). In each boxplot, the center line and extent represent the median and interquartile range (IQR; 25th–75th percentile). The whiskers extend to 1.5 × IQR. An asterisk shows the statistical significance of mean prediction accuracy (i.e., horizontal black line). RSFC = resting-state functional connectivity, AAL2 = automated anatomical labeling, group ICA = group spatial independent component analysis, RMSE = root mean square error

**Supplementary Table 1**

| Pair ID | Conversational satisfaction (Session 1) | Sex | Mean age (years) | Age gap | Department | Topic  (Session 1) |
| --- | --- | --- | --- | --- | --- | --- |
| 1 | 7.055555556 | M | 19.5 | 1 | 0 | travel |
| 2 | 5.861111111 | F | 21.5 | 1 | 0 | school life |
| 3 | 6.527777778 | F | 21 | 4 | 0 | school life |
| 4 | 5.777777778 | M | 18.5 | 1 | 0 | my hobbies |
| 5 | 5.083333333 | M | 21.5 | 1 | 0 | school life |
| 6 | 6.083333333 | M | 19.5 | 1 | 0 | school life |
| 7 | 7.111111111 | M | 19.5 | 1 | 0 | my hobbies |
| 8 | 5.416666667 | F | 20.5 | 1 | 0 | travel |
| 9 | 6.25 | F | 21 | 0 | 0 | school life |
| 10 | 7.055555556 | F | 21.5 | 1 | 0 | travel |
| 11 | 7.416666667 | F | 22.5 | 1 | 0 | travel |
| 12 | 6.666666667 | M | 21 | 2 | 0 | school life |
| 13 | 6.388888889 | F | 19.5 | 1 | 0 | school life |
| 14 | 6.222222222 | M | 22.5 | 1 | 0 | my hobbies |
| 15 | 5.805555556 | M | 22 | 2 | 1 | my hobbies |
| 16 | 7.527777778 | F | 21.5 | 1 | 1 | school life |
| 17 | 6.083333333 | F | 22 | 2 | 0 | my hobbies |
| 18 | 6.027777778 | M | 22.5 | 1 | 0 | my hobbies |
| 19 | 6.472222222 | M | 21 | 0 | 0 | school life |
| 20 | 6.388888889 | M | 21 | 0 | 0 | school life |
| 21 | 6.75 | F | 19 | 2 | 1 | travel |
| 22 | 6.444444444 | M | 22.5 | 1 | 1 | school life |
| 23 | 6.305555556 | F | 20.5 | 1 | 0 | travel |
| 24 | 5.972222222 | M | 20 | 2 | 0 | my hobbies |
| 25 | 6.388888889 | F | 19.5 | 1 | 1 | travel |
| 26 | 6.722222222 | M | 21 | 0 | 0 | travel |
| 27 | 5.972222222 | F | 22 | 0 | 0 | my hobbies |
| 28 | 6.166666667 | F | 19 | 2 | 0 | school life |
| 29 | 6.5 | M | 19 | 2 | 1 | travel |

Variables used in multiple linear regression analysis. Mean age represents age averaged across two subjects of individual pairs. Age gap represents the difference in ages of two subjects of individual pairs. Department represents whether subjects of individual pairs belong to the same department at university (0: different department, 1: same department).

**Supplementary Table 2**

Results obtained by multiple linear regression analysis to investigate influence of subjects’ demographic characteristics and conversational topics on conversational satisfaction in the first session. We applied a threshold for the results (uncorrected *p* < 0.05, two-tailed) and found no significant independent variables. The analysis had dummy variables, i.e., Sex (M: Sex = 1; F: Sex = 0), Department (same: Department = 1; different: Department = 0), and Topic (school life: Topic 1 = 1, Topic 2 = 0; travel: Topic 1 = 0, Topic 2 = 1; my hobbies: Topic 1 = 0, Topic 2 = 0).

| Independent variables | *t* value | *p* value |
| --- | --- | --- |
| Sex (M) | −0.12 | 0.91 |
| Mean age | 0.377 | 0.71 |
| Age gap | −0.00743 | 0.99 |
| Department (same) | 0.637 | 0.53 |
| Topic 1 (school life) | 0.722 | 0.48 |
| Topic 2 (travel) | 1.51 | 0.14 |

**Supplementary Table 3**

Rating for ease of talking for each topic.

| Topic | Score |
| --- | --- |
| my hobbies | 5.71428571 |
| school life | 5.48571429 |
| travel | 5.48571429 |
| likes and dislikes about foods | 5.25714286 |
| my friends | 5.2 |
| likes and dislikes about places | 4.58823529 |
| my recent events | 4.42857143 |
| likes and dislikes about seasons | 4.4 |
| likes and dislikes about books | 4.22857143 |
| likes and dislikes about video games | 4.14285714 |
| childhood play | 3.97142857 |
| my future | 3.94285714 |
| desired jobs | 3.91428571 |
| my whole life | 3.74285714 |
| my family | 3.71428571 |
| my clothes and accessories | 3.71428571 |
| my parents | 3.08571429 |
| turning points in my life | 2.62857143 |
